# Association of glycemic variability with the risk of new-onset atrial fibrillation and death in critically ill patients without diabetes: analysis of the MIMIC-IV database

**DOI:** 10.1186/s13098-025-02078-9

**Published:** 2026-01-02

**Authors:** Shengzhang Li, Jiajun Ye, Huanzhan Li, Jiahao Jiang, Yonghang Zheng, Jingxian Pei

**Affiliations:** 1 Department of Clinical Medicine, the Second Clinical School of Guangzhou Medical University, Guangzhou, 511436 China; 2 Department of Cardiology, Guangdong Key Laboratory of Vascular Diseases, Guangzhou Institute of Cardiovascular Disease, The Second Affiliated Hospital of Guangzhou Medical University, Guangzhou, 510260 China

**Keywords:** Blood glucose, Glycemic variability, Atrial fibrillation, Intensive care unit, In-hospital death

## Abstract

**Background:**

Glycemic variability (GV), a prevalent phenomenon of abnormal blood glucose levels in intensive care unit (ICU) is correlated with cardiac arrhythmias and in-hospital mortality. The specific link between GV and new-onset atrial fibrillation (AF), as well as in-hospital mortality, is not yet fully understood, particularly in critically ill patients without diabetes. Additionally, the potential mediating role of AF in this relationship has not yet been definitively established. This study investigated the association between GV, new-onset AF, and in-hospital mortality among these patients.

**Methods:**

Patients without diabetes from the Medical Information Mart for Intensive Care-IV database were included in the analysis. GV was quantified as the coefficient of variation (CV), derived from the ratio of blood glucose’s standard deviation (SD) to its mean. To evaluate the relationship between GV levels and AF incidence, plus in-hospital death, multivariate logistic regression, restricted cubic spline (RCS) analyses, and a mediation effect analysis were used.

**Results:**

The research involved 17,643 patients without diabetes, with a median age of 64, and 54.5% of them were male. The 30-day in-hospital ICU mortality rate was 14%, and the AF incidence rate was 9.7%. The adjusted logistic regression analysis revealed a significant link between log-transformed CV values and increased likelihood of atrial fibrillation (odds ratio [OR] 1.44; 95% confidence interval [95% CI] 1.32–1.56). Furthermore, this metric showed an even stronger relationship with in-hospital mortality risk (OR 2.09; 95% CI 1.94–2.24), with both findings reaching statistical significance. The RCS models indicated a potential nonlinear association between GV and these outcomes, with higher GV levels being associated with a greater probability of AF incidence and in-hospital death. The mediation analysis showed that the indirect effect of GV on in-hospital death was 36.26% after adjustment for covariates, suggesting AF may act as a mediator in the relationship between GV and in-hospital mortality.

**Conclusions:**

Elevated GV was associated with a higher incidence of new-onset AF and in-hospital mortality in non-diabetic critically ill patients, and new-onset AF may partly be a mediator of the association between GV and in-hospital mortality.

**Supplementary Information:**

The online version contains supplementary material available at 10.1186/s13098-025-02078-9.

## Background

Atrial fibrillation (AF) is one of the most common cardiac arrhythmias observed in the Intensive Care Unit (ICU), with an estimated incidence of 4%−9% in general ICU populations and even higher rates among patients with severe conditions like septic shock [1, 2]. AF frequently occurs during periods of heightened disease severity and may further exacerbate the clinical course, complicating management and increasing the risk of adverse outcomes, including thromboembolic events, major bleeding, cardiovascular complications, and all-cause mortality [1, 3–6]. Thus, new-onset AF serves as both a marker of disease severity and an independent factor for adverse outcomes. For critically ill patients, the new-onset AF is linked to risk factors like advanced age, male sex, pre-existing cardiovascular disease, and greater disease burden, such as acute respiratory failure or acute kidney injury [6–8]. However, most of these are non-modifiable in the acute care setting, highlighting the need to identify and address modifiable risk factors to improve outcomes.

Dysglycemia, which includes hyperglycemia, hypoglycemia, and glycemic variability (GV), is highly prevalent among critically ill patients, with GV being the common pattern of abnormal blood glucose in this population [9, 10]. Increasing evidence indicates that GV, rather than mean blood glucose levels, is a stronger predictor of mortality risk in ICU patients, particularly those without a history of diabetes [11, 12]. However, prior studies have linked GV to an increased risk of AF in non-critical populations, especially among individuals with type 2 diabetes, suggesting that glycemic fluctuations may promote atrial arrhythmogenesis [13–16]. Nevertheless, few investigations have been conducted to determine if this association is found in critically ill patients. Given the unique glucose metabolism pattern seen in critically ill patients compared to the general population, targeted investigations in this population are warranted.

Therefore, we conducted a retrospective study to examine the association between GV and the incidence of new-onset AF in critically ill patients without a history of diabetes, as well as to assess the impact of new-onset AF on the relationship between GV and in-hospital mortality within this cohort.

## Methods

### Study population

**Fig. 1:**
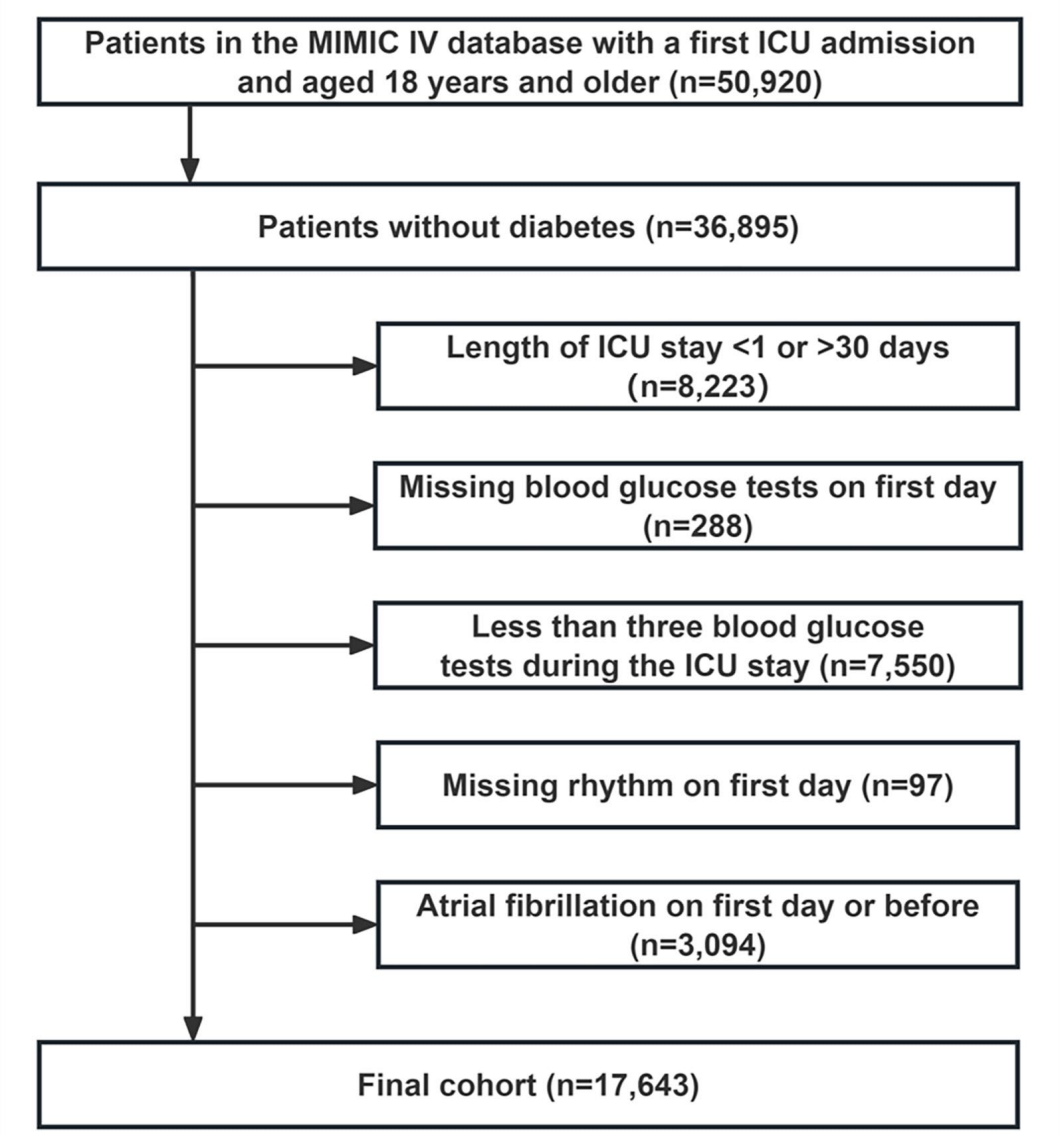
Flow of included patients through the trial

The research employed a retrospective observational approach, drawing on relevant data from the Medical Information Mart for Intensive Care IV (MIMIC-IV) database, version 2.2. The database was created through a partnership between the Beth Israel Deaconess Medical Center (BIDMC) and the Massachusetts Institute of Technology (MIT), and it included admission records from BIDMC spanning 2008 to 2019 [17]. To access the relevant data in the database, Shengzhang Li (Record ID 57075126) obtained the necessary certification and was accountable for extracting the data. Patients without diabetes were screened according to ICD-9 or ICD-10 codes as shown in Supporting file, Table S1. The exclusion criteria included: (1) patients under 18 at initial ICU admission; (2) only the first ICU admission was extracted for patients with repeated stays; (3) patients who lacked sufficient data (blood sugar and heart rate information) on the initial day of ICU admission; (4) patients with under three blood sugar tests during ICU stay; (5) patients with AF on the initial day of ICU admission or with a previous history of chronic or paroxysmal AF. Ultimately, 17,643 patients comprised the study cohort (Fig. 1).

## Data extraction

The first day in the ICU was defined as the baseline, and Navicat Premium software (version 16) was utilized to extract information from the MIMIC-IV database by executing a structured query language (SQL). The extracted information was categorized into the following seven categories: (1) Demographics, consisting of age, sex, body mass index (BMI) and race; (2) Laboratory examination, including glucose, red blood cells (RBC), white blood cells (WBC), hemoglobin, sodium, potassium, serum creatinine (Scr), and blood urea nitrogen (BUN); (3) Comorbidities, such as heart failure, myocardial infarction, cerebrovascular disease, chronic pulmonary disease, hypertension, chronic kidney disease (CKD), malignant tumor, dyslipidemia, and sepsis; (4) Disease severity score at admission, encompassing Acute Physiology ScoreⅢ(APSⅢ), Charlson’s complication score and Oxford Acute Severity of Disease Score (OASIS); (5) Vital signs, incorporating heart rate (HR), systolic blood pressure (SBP), diastolic blood pressure (DBP), and respiratory rate (RR); (6) Medication details, comprising angiotensin receptor blockers (ARB), digoxin, beta-blockers, loop diuretics (including furosemide and torasemide), angiotensin converting enzyme inhibitors (ACEI), calcium channel blockers (CCB), and oral anticoagulants (OAC, including warfarin, rivaroxaban, edoxaban and apixaban); (7) Length of hospital stay and ICU stay, ICU category, and follow-up survival status. AF was recorded as heart rhythm information in the event table of the MIMIC-IV database, and other complications were defined by the ICD-9 or ICD-10 codes as shown in Supporting file, Table S1. Concurrently, mean blood glucose levels and their standard deviation (SD) were used to determine the coefficient of variation (CV) and assess GV. The CV was calculated as the SD divided by the mean blood glucose [18] The study design considered variations in glucose sampling frequency and potential inaccuracy in AF onset timing, which might cause overlap between blood glucose measurement and AF occurrence.

## Outcomes

The primary outcome of this research was the incidence of new-onset AF after ICU admission, with 30-day mortality as a secondary outcome. And new-onset AF is defined as the first occurrence of AF during ICU admission (after 24 h) in patients with no prior documented history of AF.

### Statistical analysis

Continuous variables were described as the mean ± SD or median and interquartile range (IQR), while categorical variables were presented as numbers and percentages(%). The analysis of normally distributed continuous variables was performed using a t-test or ANOVA, while the Mann-Whitney U test or Kruskal-Wallis test was used for continuous variables that were not normally distributed. Fisher’s exact test or Pearson’s chi-square test was utilized for comparing categorical variables between groups.

Given the skewed distribution of CV, a natural logarithmic transformation was applied to normalize the data distribution, and stratified by quartiles of CV. The multivariate logistic regression analysis was used to compare the association between GV and the outcomes of the study in the different groups according to CV quartiles(Supporting file, Table S3 and Table S4). A logistic regression was utilized to determine the relationship between GV and the outcomes of the study [18, 19]. The covariates were gender, age, race, Body Mass Index(BMI), ICU category, heart rate, respiratory rate, Laboratory indicators (WBC, hemoglobin, Scr, potassium, and sodium), comorbidities(chronic pulmonary disease, cerebrovascular disease, hypertension, heart failure, chronic kidney disease, dyslipidemia, malignant cancer and Sepsis), medications (ACEI, ARB, beta blocker, digoxin, loop diuretics, CCB, OAC, statin, and antiplatelet), and disease severity scores(Charlson, APSⅢ, and OASIS). Next, a restricted cubic spline (RCS) was used to illustrate potential non-linear associations between GV and the outcomes. Further, a structural equation model (SEM) was used to determine whether new-onset AF partially mediated the relationship between GV and in-hospital mortality. Moreover, to evaluate the robustness of AF as a mediator between GV and in-hospital mortality across different subgroups of clinical characteristics, we conducted a Subgroup Mediation Analysis. The analysis was performed using a SEM framework. Given that both the mediator variable (AF) and the outcome variable (in-hospital mortality) were binary variables, we utilized the Weighted Least Squares Mean and Variance adjusted (WLSMV) estimator, which demonstrates good robustness in handling categorical data and non-normal distributions. Finally, subgroup analyses were performed based on predefined categories such as age(< 65 or ≥ 65 years), gender, race, BMI(< 30 or ≥ 30), CKD, AMI, heart failure, hypertension, dyslipidemia, chronic pulmonary disease, cerebrovascular disease, sepsis and malignant cancer.

All data analyses were performed using R statistical package (R version 4.4.2) and *P* < 0.05 indicated statistical significance.

## Results

### Baseline characteristics

**Fig. 2:**
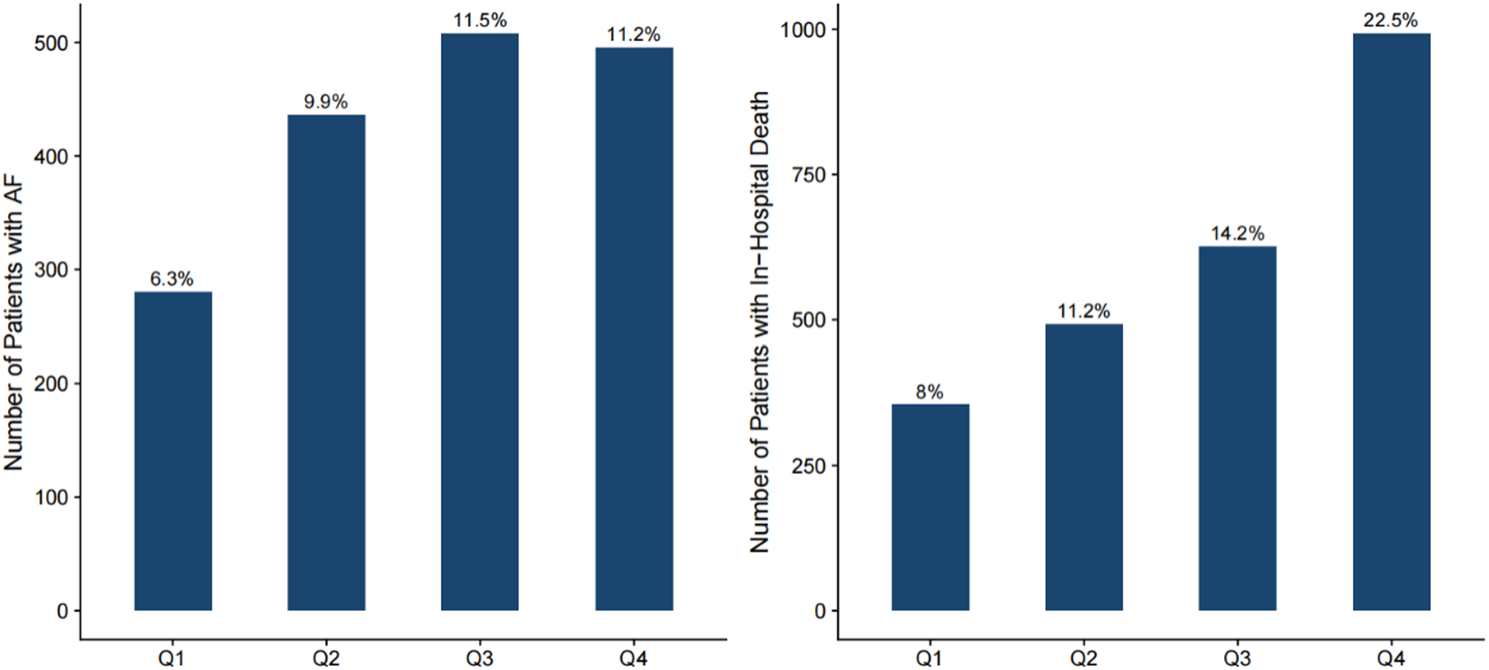
Incidence of new onset AF and in-hospital mortality in the four groups according to CV quartiles This bar chart depicts the distribution of clinical outcomes among hospitalized patients stratified by quartiles Q1–Q4. The left panel represents the number of patients diagnosed with atrial fibrillation (AF), while the right panel illustrates the number of in-hospital deaths. Bar heights indicate absolute case counts for each category, with corresponding proportions explicitly annotated. Abbreviations: AF, atrial fibrillation

A total of 17,643 patients were enrolled in the study. Their median age of the patients was 64 years, 54.5% were male and 24% were admitted to the Cardiac Care Unit (CCU). The baseline characteristics of the patients divided into four groups according to CV quartiles (quartile Q1: <11.54; Q2: 11.54—17.08; Q3: 17.08—24.40; Q4: >24.40) were shown in the Table 1.Patients with a higher CV exhibited the higher levels of WBC, initial blood glucose, mean blood glucose, RR, BUN, Scr, higher illness severity scores (APSⅢ, OASIS), and lower BMI, hemoglobin, and SBP. Notably, those with higher CV were more likely to have comorbidities, such as myocardial infarction, heart failure, chronic renal failure, and sepsis, as well as higher rates of in-hospital mortality and AF. These findings were also shown in Fig. 2, illustrating the correlation between high GV and an increased incidence of new-onset AF and in-hospital mortality.

**Table 1:** Baseline characteristics of the study population according to quartiles of glycemic variability

Variables	Overall(*N* = 17643)	Q1(*N* = 4411) (CV:<11.54)	Q2(*N* = 4410) (CV:11.54—17.08)	Q3(*N* = 4412) (CV:17.08—24.40)	Q4(*N* = 4410) (CV:>24.40)	*P* value
Age, year, (median[IQR])	64.0 (51.0 to 76.0)	62.0 (49.0 to 75.0)	63.0 (50.0 to 75.0)	64.0 (52.0 to 76.0)	64.0 (52.0 to 77.0)	< 0.001
Gender, *n*(%)						< 0.001
Female	8026 (45.5%)	1860 (42.2%)	1923 (43.6%)	2034 (46.1%)	2209 (50.1%)	
Male	9617 (54.5%)	2551 (57.8%)	2487 (56.4%)	2378 (53.9%)	2201 (49.9%)	
Race, n(%)						0.002
Asian	519 (2.9%)	118 (2.7%)	125 (2.8%)	123 (2.8%)	153 (3.5%)	
Black	1358 (7.7%)	330 (7.5%)	305 (6.9%)	358 (8.1%)	365 (8.3%)	
Other	1351 (7.7%)	344 (7.8%)	347 (7.9%)	325 (7.4%)	335 (7.6%)	
Unknown	2630 (14.9%)	597 (13.5%)	662 (15%)	651 (14.8%)	720 (16.3%)	
White	11,785 (66.8%)	3022 (68.5%)	2971 (67.4%)	2955 (67%)	2837 (64.3%)	
Weight, kg, (median[IQR])	77.2 (64.8 to 91.3)	79.7 (66.5 to 93.2)	78.0 (66.0 to 92.4)	76.6 (64.4 to 91.3)	74.5 (62.1 to 88.5)	< 0.001
Height, cm, (median[IQR])	169.3 (168.0 to 170.0)	169.3 (169.3 to 170.0)	169.3 (168.0 to 173.0)	169.3 (168.0 to 173.0)	169.3 (165.0 to 170.0)	< 0.001
BMI, kg/m2,(median[IQR])	26.9 (23.0 to 31.4)	27.4 (23.5 to 32.0)	27.1 (23.3 to 31.6)	26.8 (22.9 to 31.1)	26.1 (22.3 to 30.6)	< 0.001
ICU, n(%)						0.005
CCU	4227 (24%)	1136 (25.8%)	1065 (24.1%)	1016 (23%)	1010 (22.9%)	
Other	13,416 (76%)	3275 (74.2%)	3345 (75.9%)	3396 (77%)	3400 (77.1%)	
Vital Signs						
HR, (median [IQR])	83.3 (73.5 to 95.4)	81.6 (72.1 to 92.3)	83.0 (73.5 to 95.2)	83.8 (73.6 to 95.5)	85.5 (75.0 to 98.1)	< 0.001
SBP, mmHg, (median[IQR])	117.4 (105.7 to 126.7)	117.4 (107.8 to 129.3)	117.4 (106.0 to 126.9)	116.8 (105.1 to 126.0)	114.9 (103.9 to 123.7)	< 0.001
DBP, mmHg, (median[IQR])	65.5 (58.0 to 71.6)	65.5 (59.2 to 73.6)	65.5 (58.4 to 71.5)	65.5 (57.6 to 71.0)	64.9 (56.8 to 70.2)	< 0.001
RR, (median [IQR])	18.5 (16.4 to 21.2)	18.1 (16.2 to 20.5)	18.4 (16.4 to 21.1)	18.5 (16.4 to 21.3)	18.9 (16.6 to 22.0)	< 0.001
Comorbidities, n (%)						
Chronic pulmonary disease	649 (3.7%)	152 (3.4%)	159 (3.6%)	158 (3.6%)	180 (4.1%)	0.41
Cerebrovascular disease	2933 (16.6%)	823 (18.7%)	825 (18.7%)	743 (16.8%)	542 (12.3%)	< 0.001
Hypertension	7285 (41.3%)	1833 (41.6%)	1871 (42.4%)	1852 (42%)	1729 (39.2%)	0.011
Myocardial infarction	2346 (13.3%)	506 (11.5%)	586 (13.3%)	597 (13.5%)	657 (14.9%)	< 0.001
Heart failure	3316 (18.8%)	705 (16%)	788 (17.9%)	873 (19.8%)	950 (21.5%)	< 0.001
Chronic kidney disease	2190 (12.4%)	475 (10.8%)	485 (11%)	578 (13.1%)	652 (14.8%)	< 0.001
Dyslipidemia	5755 (32.6%)	1499 (34%)	1426 (32.3%)	1460 (33.1%)	1370 (31.1%)	0.027
Malignant cancer	1090 (6.2%)	256 (5.8%)	257 (5.8%)	267 (6.1%)	310 (7%)	0.054
Sepsis	9543 (54.1%)	1732 (39.3%)	2362 (53.6%)	2619 (59.4%)	2830 (64.2%)	< 0.001
Charlson, (median [IQR])	4.0 (2.0 to 6.0)	4.0 (2.0 to 6.0)	4.0 (2.0 to 6.0)	4.0 (2.0 to 6.0)	4.0 (2.0 to 6.0)	< 0.001
Laboratory tests						
WBC, K/uL, (median [IQR])	11.1 (8.2 to 14.8)	10.3 (7.8 to 13.8)	11.0 (8.2 to 14.6)	11.3 (8.4 to 15.2)	11.6 (8.3 to 15.9)	< 0.001
RBC, m/uL, (median [IQR])	3.6 (3.1 to 4.2)	3.7 (3.2 to 4.2)	3.7 (3.2 to 4.2)	3.6 (3.1 to 4.1)	3.6 (3.1 to 4.1)	< 0.001
Hemoglobin, g/dL, (median[IQR])	11.0 (9.5 to 12.6)	11.3 (9.7 to 12.8)	11.1 (9.6 to 12.7)	10.9 (9.4 to 12.5)	10.7 (9.3 to 12.4)	< 0.001
Initialbloodglucose, mg/dL, (median[IQR])	122.3 (105.3 to 143.7)	112.5 (100.5 to 126.7)	120.0 (106.0 to 136.3)	126.0 (107.2 to 145.5)	141.0 (114.5 to 173.7)	< 0.001
Meanbloodglucose, mg/dL, (median[IQR])	120.5 (107.2 to 136.0)	112.0 (100.3 to 124.8)	118.0 (107.0 to 130.4)	122.3 (109.5 to 135.7)	133.0 (116.4 to 156.7)	< 0.001
Potassium, mEq/L, (median [IQR])	4.1 (3.8 to 4.4)	4.1 (3.8 to 4.4)	4.1 (3.8 to 4.4)	4.1 (3.8 to 4.5)	4.1 (3.8 to 4.5)	< 0.001
Sodium, mEq/L, (median [IQR])	138.8 (136.3 to 141.0)	139.0 (136.5 to 141.0)	138.8 (136.3 to 141.0)	138.7 (136.0 to 141.0)	138.7 (136.0 to 141.1)	0.491
BUN, mg/dL, (median [IQR])	16.5 (11.5 to 25.0)	15.0 (11.0 to 22.0)	16.0 (11.5 to 23.0)	17.0 (11.8 to 26.4)	19.0 (12.5 to 30.5)	< 0.001
SCR, mg/dL, (median [IQR])	0.9 (0.7 to 1.2)	0.8 (0.7 to 1.1)	0.9 (0.7 to 1.2)	0.9 (0.7 to 1.3)	1.0 (0.7 to 1.5)	< 0.001
Medications, n(%)						
ACEI	2617 (14.8%)	675 (15.3%)	686 (15.6%)	660 (15%)	596 (13.5%)	0.033
ARB	763 (4.3%)	226 (5.1%)	196 (4.4%)	178 (4%)	163 (3.7%)	0.007
Betablocker	9139 (51.8%)	2189 (49.6%)	2418 (54.8%)	2376 (53.9%)	2156 (48.9%)	< 0.001
CCB	2268 (12.9%)	573 (13%)	683 (15.5%)	610 (13.8%)	402 (9.1%)	< 0.001
Digoxin	204 (1.2%)	30 (0.7%)	47 (1.1%)	66 (1.5%)	61 (1.4%)	0.001
Loopdiuretics	8009 (45.4%)	1530 (34.7%)	2024 (45.9%)	2270 (51.5%)	2185 (49.5%)	< 0.001
OAC	2460 (13.9%)	638 (14.5%)	621 (14.1%)	640 (14.5%)	561 (12.7%)	0.052
Statin	1590 (9%)	410 (9.3%)	413 (9.4%)	406 (9.2%)	361 (8.2%)	0.174
Antiplatelet	5823 (33%)	1445 (32.8%)	1506 (34.1%)	1472 (33.4%)	1400 (31.7%)	0.104
APSIII, (median [IQR])	38.0 (29.0 to 52.0)	33.0 (25.0 to 43.0)	37.0 (28.0 to 49.0)	40.0 (30.0 to 53.0)	45.0 (34.0 to 62.0)	< 0.001
OASIS, (median [IQR])	31.0 (25.0 to 37.0)	28.0 (23.0 to 34.0)	30.0 (25.0 to 36.0)	31.0 (26.0 to 37.0)	33.0 (27.0 to 39.0)	< 0.001
Length of stay(LOS)						
LOS in hospital, day, (median[IQR])	8.0 (5.0 to 13.8)	6.8 (4.4 to 10.6)	8.7 (5.5 to 14.4)	8.8 (5.6 to 15.8)	8.2 (5.0 to 14.6)	< 0.001
LOS in ICU, day, (median [IQR])	3.0 (2.0 to 5.2)	2.4 (1.8 to 3.6)	3.2 (2.1 to 5.9)	3.5 (2.1 to 6.5)	3.2 (2.0 to 5.8)	< 0.001

Categorical variables are presented as n (%) and continuous variables are presented as mean (standard deviation). *p* < 0.05 is taken as statistical significance. Abbreviations: AF, atrial fibrillation; ICU, intensive care unit; CCU, coronary care unit; BMI, body mass index; HR, heart rate; RR, respiratory rate; BP, blood pressure; SBP, systolic blood pressure; DBP, diastolic blood pressure; WBC, white blood cell; RBC, red blood cell; BUN, blood urea nitrogen; SCR, serum creatinine; ACEI, angiotensin converting enzyme inhibitor; ARB, angiotensin receptor blockers; CCB, calcium channel blocker; OAC, oral anticoagulant; APSⅢ, Acute Physiology ScoreⅢ; OASIS, oxford acute severity of illness score; LOS, length of stay

Supporting file, Table S2 presented the baseline characteristics of the patients with new-onset AF and those without new-onset AF during hospitalization. Patients with new-onset AF were generally older and more likely to have comorbidities, such as sepsis, hypertension, heart failure, dyslipidemia, myocardial infarction, chronic renal failure, and chronic lung disease (all *P* < 0.001). Notably, 73% of new-onset AF cases were patients with sepsis. They also had higher levels of WBC, initial blood glucose, mean blood glucose, blood potassium, BUN, Scr, GV, BMI, and disease severity scores (APSⅢ and OASIS). Moreover, patients in the new-onset AF group were more frequently prescribed β-blockers, digoxin, loop diuretics, OAC, statins, and antiplatelet agents, and had longer hospital and ICU stays. Similar to patients with high GV in Fig. 2, new-onset AF cases showed higher in-hospital mortality (*P* < 0.001).

### Glycemic variability linked to new-onset AF and in-hospital death

Multivariate logistic regression models showed that log-transformed CV (LogCV) was positively associated with a higher odds of new-onset AF (OR 1.44; 95% CI 1.32–1.56; *P* < 0.001) and in-hospital death (OR 2.09; 95% CI 1.94–2.24; *P* = 0.002) (Table 2). The adjusted logistic model showed a 24% increase in the risk of new-onset AF (OR 1.24; 95% CI 1.11–1.38; *P* < 0.001) and a 15% increase in the risk of in-hospital death (OR 1.15; 95% CI 1.05–1.26; *P* = 0.003) (Table 2). Although the OR decreased compared to the crude model, it was still statistically different, indicating that GV remained significantly associated with adverse outcomes.

**Table 2:** Association between glucose variability and New-Onset AF and In-Hospital mortality

Outcomes	Crude OR Per unit of Log CV	Crude *P* value	Adjusted OR Per unit of Log CV	Adjusted *P* value
	Model 1		Model 2	
New-onset AF	1.44(1.32–1.56)	< 0.001	1.24(1.11–1.38)	< 0.001
In-hospital death	2.09(1.94–2.24)	< 0.001	1.15(1.05–1.26)	0.003
New-onset AF and in-hospital death	2.51(2.14–2.94)	< 0.001	1.57(1.29–1.92)	< 0.001

Model 1: unadjusted;

Model 2: adjusted for gender, age, race, BMI, ICU category, heart rate, respiratory rate, WBC, hemoglobin, Scr, potassium, sodium, chronic pulmonary disease, cerebrovascular disease, hypertension, heart failure, chronic kidney disease, dyslipidemia, malignant cancer, sepsis, medications and disease severity scores(Charlson, APSⅢ, and OASIS)

Abbreviations: AF, atrial fibrillation; OR, odds ratio; BMI, body mass index; ICU, intensive care unit; WBC, white blood cells; BUN, blood urea nitrogen; Scr, serum creatinine; APSⅢ, autoimmune polyendocrine syndrome typeⅢ; OASIS, oxford acute severity of illness score

**Fig. 3:**
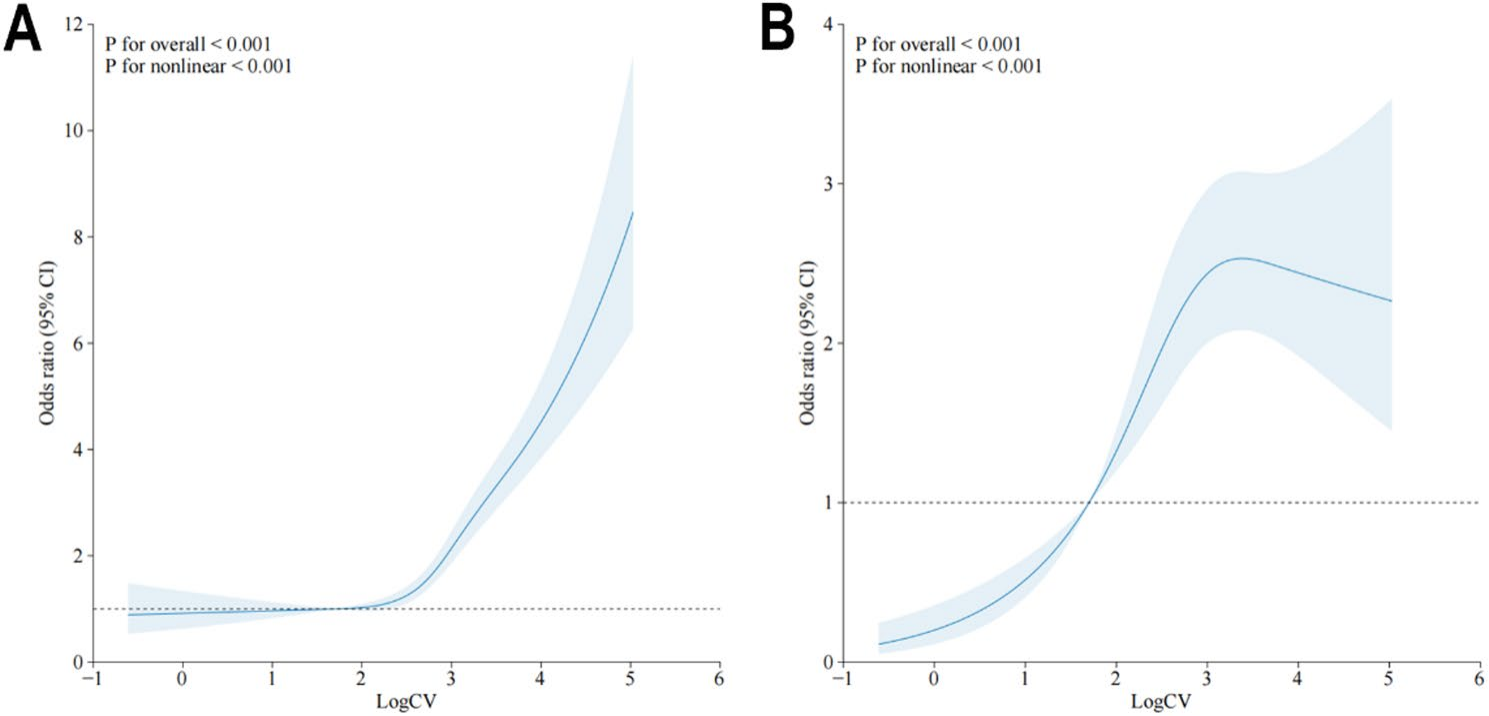
RCS of the association of glycemic variability with the occurrence of in-hospital death (**A**) and AF (**B**) The central line in bold represents the estimated adjusted hazard ratio, while the shaded area indicates the 95% confidence interval. Abbreviations: RCS, Restricted cubic splines; AF, atrial fibrillation

The RCS model was utilized to illustrate a potential non-linear association between LogCV and the outcomes, with race and sex as moderating factors. The results of RCS revealed the non-linear association between LogCV and the outcomes (p for non-linearity < 0.001). As shown in Fig. 3, the association between LogCV and in-hospital mortality exhibited a roughly linear increase. In contrast, the relationship between LogCV and new-onset AF presented an inverted U-shape. Specifically, within the LogCV range of 1 to 3, a positive association was observed, indicating that increased glycemic variability was linked to higher AF risk. However, in higher LogCV intervals, the odds ratios (ORs) demonstrated a plateauing trend, suggesting that the impact of glycemic fluctuations on AF risk gradually attenuated.

### Subgroup analysis

**Fig. 4:**
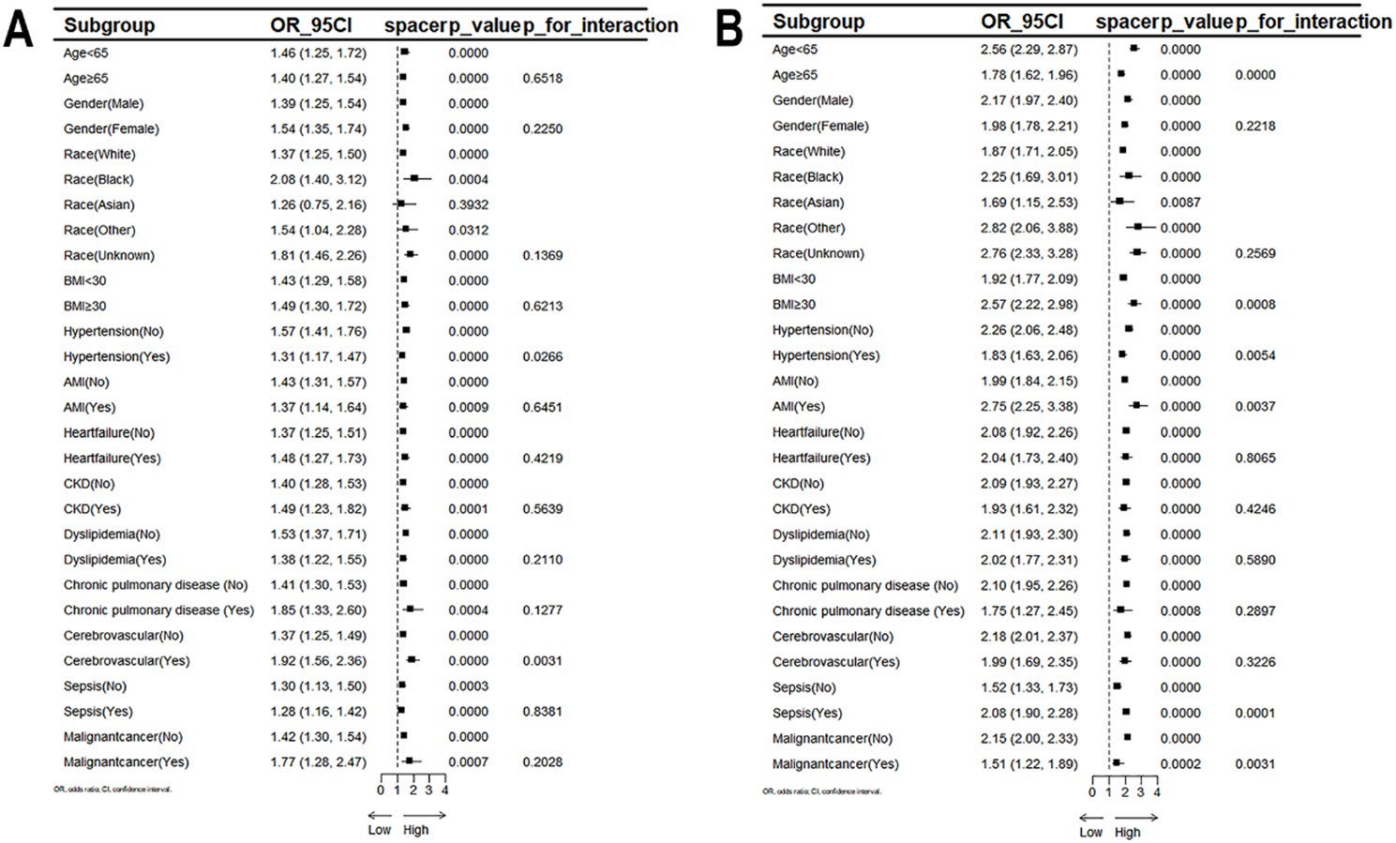
Subgroup analyses of glycemic variability associated with AF (**A**) and in-hospital mortality (**B**) The subgroup analysis was adjusted for age, BMI, gender, comorbidities and race. *p* < 0.050 is taken as statistical significance. Abbreviations: OR, odds ratio; CI, confidence interval; AF, atrial fibrillation; BMI, body mass index; AMI, acute myocardial infarction; CKD, chronic kidney disease

To assess the impact of GV on AF and mortality during hospitalization, participants were categorized into subgroups for subgroup analysis according to different characteristics (including age, BMI, sex, ethnicity, and comorbidities). Our stratified analysis of GV and AF risk showed a positive association with AF occurrence in all subgroups except Asians (all *P* < 0.05), as shown in Fig. 4. Subgroup analyses showed a strong, positive link with in-hospital mortality across all categories (all *P* < 0.01).

Meanwhile, the interaction analysis showed that in the subgroup analysis with the occurrence of new-onset AF as the outcome event, a notable interaction was observed between GV and whether hypertension or cerebrovascular disease was present or absent (P for interaction < 0.05)(Fig. 4), whereas the age bracket, BMI category, whether or not hypertension was present, the occurrence of acute myocardial infarction, the presence of sepsis, and the existence of malignancy all played a pivotal role in the correlation between GV and the likelihood of in hospital death(P for interaction < 0.01), Fig. 4.

### AF may partly mediate the association between GV and in-hospital death

**Fig. 5:**
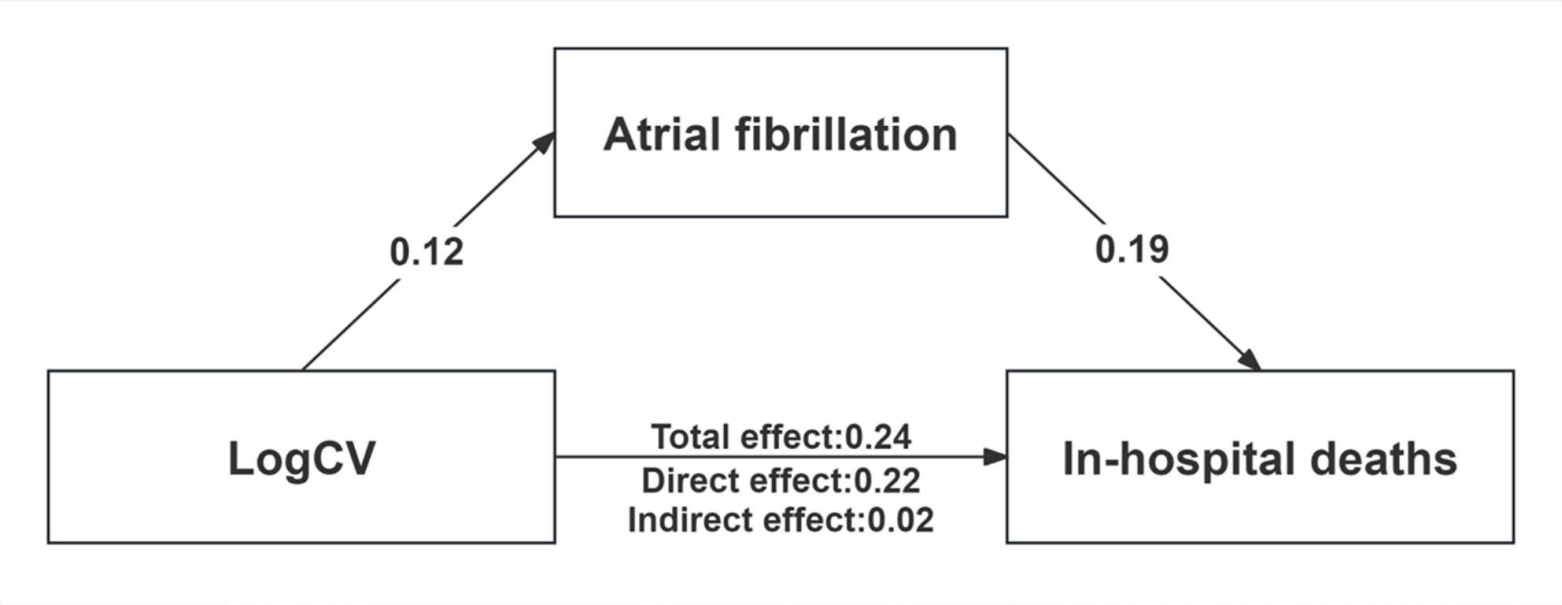
Direct and indirect effects (mediated by AF) of glycemic variability on hospital deaths. Abbreviation: AF, atrial fibrillation

The results of the mediation analysis were showed in Fig. 5. In the crude model, the proportion of indirect effects to the total effect was approximately 9%, suggesting a possible mediating role of AF in the association between GV and in-hospital death (*P* < 0.001 for indirect effect). Notably, after adjusting for covariates, the proportion of GV on in-hospital mortality rose to 36.26%, indicating a stronger potential mediation pathway through the new-onset AF (*P* < 0.001 for indirect effect, Supporting file, Table S5).

The results of the subgroup analysis indicated that the interaction tests were generally non-significant (P for interaction > 0.05, Supporting file, Table S6) across most subgroups (e.g., age, gender, presence of hypertension, myocardial infarction, heart failure, dyslipidemia, Chronic pulmonary disease, cerebrovascular disease, and malignant cancer.). This suggested that the risk mechanism where elevated GV leads to mortality through AF was consistently stable regardless of whether patients have these comorbidities. The analysis also revealed a significant interaction in the sepsis group (P for interaction = 0.028). In septic patients, this mediation pathway was highly significant (OR = 1.01, 95% CI: 1.01–1.02, *P* < 0.001), however, it was not significant in non-septic patients (OR = 1.00, *P* = 0.520). This finding suggested that sepsis may play a critical modifying role in the mediation effect.

## Discussion

This retrospective study, based on a large, public-accessible MIMIC-IV database, evaluated 17,643 critically ill patients without diabetes, and a total of 1719 patients (9.7%) developed new-onset AF. To our understanding, this was the first research involving a large sample size on this clinical subject. Our results indicated that LogCV, an indicator of GV, was associated with increased AF risk and higher in-hospital death rates in critically ill patients without diabetes (*P* < 0.001, all crude and adjusted, Table 2).

Previous studies involving ICU similarly indicated the correlation between GV and AF mainly focused on cardiac surgical patients. Sim et al. [20] showed that increased magnitudes of acute perioperative glycemic fluctuations increased the length of ICU stay in patients undergoing coronary artery bypass grafting (CABG) and risk of postoperative atrial fibrillation (POAF). A retrospective study also indicated that increased GV was associated with a higher incidence of POAF in patients undergoing various cardiac surgeries, such as CABG, aortic, and heart valve procedures [15]. Furthermore, the effect of glycemic fluctuations on AF has also been studied in mouse models with diabetes. Saito et al. [21] triggered glycemic fluctuations via a 24-hour fast and subsequent insulin infusions, noting that raised GV elevated atrial fibrillation prevalence.

GV has emerged as a potential contributor to AF by promoting accelerated cardiac structural and electrical remodeling. A previous study demonstrated that glycemic fluctuations intensified myocardial fibrosis, increasing susceptibility to atrial fibrillation [22]. In addition to structural remodeling, a basic study showed that hyperglycemia led to significant changes in atrial electrical activity, manifested as a shortening of atrial effective refractory period (AERP) and an increase in Atrial Effective Refractory Period Dispersion (AERPD), due to the increase of L-type calcium current density and the density of sodium current decreased [23]. Hegy et al. [24] demonstrated that even though acute hyperglycaemia alone could increase cellular proarrhythmic mechanisms and the presence of additional pathological factors (i.e., heart failure, hypo-and hyperkalaemia) could further increase the arrhythmogenic effects, which may support our finding that high GV increased the risk of NOAF in the condition of critical illness. Inflammation and oxidative stress may help explain the association between GV and new-onset AF during critical illness. Wu and colleagues [25] developed an animal model for acute fluctuating hyperglycemia and discovered that such glycemic fluctuations can lead to notable oxidative stress and inflammation in endothelial cells. Saito et al. [21] also demonstrated that in comparison to the controls, DM rats with glycemic fluctuations had significantly increased levels of reactive oxygen species (ROS). Moreover, the research also pointed out that elevated ROS may be implicated in cardiac fibrosis resulting from glycemic fluctuations. Although our study did not investigate the effect of GV on the biomarkers of ROS and inflammation, prior clinical studies had also confirmed that increased GV was positively linked to higher levels of oxidative stress [26, 27].

Our findings also indicated that elevated GV showed a significant association with increased mortality rates among critically ill patients (*P* < 0.001), consistent with the findings of most previous studies. A multicenter retrospective study by Egi and Krinsley demonstrated that GV was a critical and independent predictor of ICU and in-hospital mortality, even surpassing mean blood glucose levels in predictive strength [11, 12]. Bagshaw et al. [28] defined GV as the occurrence of hypoglycemic and hyperglycemic episodes within 24 h of ICU admission and identified its strong association with ICU mortality. Furthermore, Hsu et al. [29] examined the effect of GV on ICU and in-hospital death in patients with diabetes and without diabetes. Meanwhile, they found that patients without diabetes with high GV had the highest mortality rates. Similarly, Krinsley et al. [30] also indicated that GV exerted a greater impact on critically ill patients without diabetes, whereas the effects of GV on patients with diabetes were relatively less pronounced. This may be because critically ill patients with diabetes could tolerate greater glycemic fluctuations than patients without diabetes. However, no studies reported the link between glucose variability, new-onset AF and the risk of in-hospital death. Our study suggested that the association between GV and in-hospital mortality may be partly explained by new-onset AF. We also found that the mechanism by which GV increased mortality risk via inducing AF was more specific and sensitive in septic patients. This may be attributed to the high inflammatory state of sepsis patients and the atrial electrical activity being more susceptible to glycemic fluctuations. In addition to its association with cardiovascular complications such as AF, GV was also linked to other adverse outcomes, including neurological injury and infections [31–33]. These findings indicated that GV may contribute to multi-organ dysfunction through mechanisms involving oxidative stress, inflammation, apoptosis, and endothelial injury. This was consistent with the finding of Klimontov et al., who proposed that GV may alter gene expression profiles and modulate intracellular signaling pathways, thereby adversely affecting the life cycle and function of target cells in the nervous system, kidneys, liver, and other organs [34]. These mechanisms may elevate the risk of death among critically ill patients. This may also help explain another finding from the subgroup analysis that patients with comorbidities such as acute myocardial infarction, sepsis, or malignancies showed a more pronounced increase in ICU mortality risk compared with those without these conditions. Moreover, the integration of GV, AF onset, and mortality risk in critically ill patients could occur through mechanisms involving enhanced oxidative stress, endothelial dysfunction, and the activation of pro-inflammatory pathways.

There were some limitations in the study. First, based on the retrospective nature of this study, multiple confounders could not be fully avoided. Second, for repeat ICU admissions, we only analyzed the baseline characteristics of the first ICU admission and only included patients with more than three blood glucose measurements, which may also have contributed to a selection bias. Third, we excluded patients with a previous history of AF and an episode of AF on the first day of ICU admission, but it is possible that patients may have had previous AF without knowing it, due to no symptoms. Fourthly, the blood glucose measurements were not standardized for every patient, which may lead to some deviation in the results. Furthermore, we calculated GV based on all glucose values during ICU stay. While this approach may better reflect overall GV and reduce errors from infrequent measurements, it may inadvertently incorporate post-AF GV, confounding their correlation analysis. Fifth, the limitations of this study included variability in glucose sampling frequency and imprecise determination of AF onset times, potentially leading to data overlap and compromising the accuracy of the results. Sixth, despite comprehensive multivariable adjustments, several biologically plausible ICU variables (e.g., vasopressors, insulin therapy and mechanical ventilation) were excluded due to > 20% missing data, potentially introducing residual confounding. Finally, cardiac rhythm information was recorded on the medical record form, and the frequency and severity of AF were difficult to assess.

## Conclusions

Glycemic variability was associated with higher odds of new-onset AF and in-hospital mortality among critically ill patients without diabetes, and new-onset AF may partly mediate this association.

## Supplementary Information

Supplementary Material 1

## Data Availability

No datasets were generated or analysed during the current study.

## Abbreviations

GV: Glycemic variability
AF: Atrial fibrillation
MIMIC-IV: Medical Information Mart for Intensive Care
CV: Coefficient of variation
RCS: Restricted cubic spline
ICU: Intensive care unit
BIDMC: Beth Israel Deaconess Medical Center
MIT: Massachusetts Institute of Technology
SQL: Structured Query Language
BMI: Body mass index
RBC: Red blood cell
WBC: White blood cell
BUN: Blood urea nitrogen
Scr: Serum creatinine
CKD: Chronic kidney disease
APSIII: Acute Physiology Score III
OASIS: Oxford Acute Severity of Illness Score
HR: Heart rate
RR: Respiratory rate
SBP: Systolic blood pressure
DBP: Diastolic blood pressure
ARB: Angiotensin receptor blockers
ACEI: Angiotensin converting enzyme inhibitors
CCB: Calcium channel blockers
OAC: Oral anticoagulants
SD: Standard deviation
AMI: Acute myocardial infarction
CCU: Coronary Care Unit
LogCV: Log-transformed coefficient of variation
CABG: Coronary artery bypass grafting
POAF: Postoperative atrial fibrillation
AERP: Atrial effective refractory period
AERPD: Atrial Effective Refractory Period Dispersion
ICa: L-type calcium current
INa: Sodium current
ROS: Reactive oxygen species
