# Supplementary material for: Association of glycemic variability with the risk of new-onset atrial fibrillation and death in critically ill patients without diabetes: analysis of the MIMIC-IV database
Source: Diabetol Metab Syndr. 2026 Jan 2;18:43. doi: 10.1186/s13098-025-02078-9 (PMC12866588; doi:10.1186/s13098-025-02078-9)
Supplement: Supplementary file 1 — Supplementary Material 1 [file 13098_2025_2078_MOESM1_ESM.doc]

**Supporting File**

**Supporting file, Table S1 Disease diagnosis of this study and corresponding ICD codes.**

| Disease diagnosis | ICD-9 codes | ICD-10 codes |
| --- | --- | --- |
| Heart failure | 428, 39891, 40201, 40211, 40291, 40401, 40403, 40411, 40413, 40491, 40493, 4254, 4259 | I43, I50, I099, I110, I130, I132, I255, I420, I425-I429, P290 |
| Myocardial infarction | 410, 412 | I21, I22, I252 |
| Cerebrovascular disease | 430-438 | I60-I69 |
| Chronic pulmonary disease | 490, 505, 4168, 4169, 5064, 5081, 5088 | J40, J47, J60, J67, I278, I279, J684, J701, J703 |
| Hypertension | 4010, 4011, 4019, 40501, 40509, 40511, 40519, 40591, 40599, 64211-64214 | I10, I15, I150-I152, I158, I159 |
| Diabetes | 2500-2509 | E100-E149 |
| Chronic kidney disease | 585-586 | N18 |
| Malignant cancer | 140, 172, 1740, 1958, 200, 208, 2386 | C43, C88, C00-C26, C30-C34, C37-C41, C45-C58, C60-C76, C81-C85, C90-C97 |
| Dyslipidemia | 2720-2729 | E78, E780-E789, E7800-E7801, E7841, E7849, E7870-E7872, E7879, E7881, E7889 |
| Cerebral infarction | 43301, 43311, 43321, 43331, 43381, 43391, 43401, 43411 | I63 |

**Supporting file, Table S2 Baseline characteristics of the study population according to whether or not atrial fibrillation occurred**

| **Variables** | **Overall (N=17643)** | **non-AF (N=15924)** | **AF (N=1719)** | **P value** |
| --- | --- | --- | --- | --- |
| **Age,year,(median[IQR])** | **64.0 (51.0 to 76.0)** | **62.0 (49.0 to 74.0)** | **74.0 (65.0 to 83.0)** | **<.001** |
| **Gender,n(%)** |  |  |  | **0.001** |
| **Female** | **8026 (45.5%)** | **7309 (45.9%)** | **717 (41.7%)** |  |
| **Male** | **9617 (54.5%)** | **8615 (54.1%)** | **1002 (58.3%)** |  |
| **Race,n(%)** |  |  |  | **<.001** |
| **Asian** | **519 (2.9%)** | **481 (3%)** | **38 (2.2%)** |  |
| **Black** | **1358 (7.7%)** | **1293 (8.1%)** | **65 (3.8%)** |  |
| **Other** | **1351 (7.7%)** | **1277 (8%)** | **74 (4.3%)** |  |
| **Unknown** | **2630 (14.9%)** | **2378 (14.9%)** | **252 (14.7%)** |  |
| **White** | **11785 (66.8%)** | **10495 (65.9%)** | **1290 (75%)** |  |
| **Weight,kg,(median[IQR])** | **77.2 (64.8 to 91.3)** | **77.0 (64.7 to 91.0)** | **79.3 (65.2 to 94.7)** | **<.001** |
| **Height,cm,(median[IQR])** | **169.3 (168.0 to 170.0)** | **169.3 (168.0 to 170.0)** | **169.3 (165.0 to 175.0)** | **0.062** |
| **BMI,kg/m2,(median[IQR])** | **26.9 (23.0 to 31.4)** | **26.8 (22.9 to 31.3)** | **27.5 (23.4 to 32.0)** | **<.001** |
| **ICU,n(%)** |  |  |  | **<.001** |
| **CCU** | **4227 (24%)** | **3375 (21.2%)** | **852 (49.6%)** |  |
| **Other** | **13416 (76%)** | **12549 (78.8%)** | **867 (50.4%)** |  |
| **Vital Signs** |  |  |  |  |
| **HR,(median [IQR])** | **83.3 (73.5 to 95.4)** | **83.6 (73.4 to 95.8)** | **81.5 (74.5 to 90.5)** | **<.001** |
| **SBP,mmHg,(median[IQR])** | **117.4 (105.7 to 126.7)** | **117.4 (105.9 to 127.2)** | **117.3 (104.3 to 118.3)** | **<.001** |
| **DBP,mmHg,(median[IQR])** | **65.5 (58.0 to 71.6)** | **65.5 (58.2 to 72.2)** | **64.7 (55.6 to 65.5)** | **<.001** |
| **RR,(median [IQR])** | **18.5 (16.4 to 21.2)** | **18.5 (16.4 to 21.2)** | **18.6 (16.5 to 21.4)** | **0.073** |
| **Comorbidities,n (%)** |  |  |  |  |
| **Chronic pulmonary disease** | **649 (3.7%)** | **530 (3.3%)** | **119 (6.9%)** | **<.001** |
| **Cerebrovascular disease** | **2933 (16.6%)** | **2630 (16.5%)** | **303 (17.6%)** | **0.254** |
| **Hypertension** | **7285 (41.3%)** | **6452 (40.5%)** | **833 (48.5%)** | **<.001** |
| **Myocardial infarction** | **2346 (13.3%)** | **1967 (12.4%)** | **379 (22%)** | **<.001** |
| **Heart failure** | **3316 (18.8%)** | **2726 (17.1%)** | **590 (34.3%)** | **<.001** |
| **Chronic kidney disease** | **2190 (12.4%)** | **1876 (11.8%)** | **314 (18.3%)** | **<.001** |
| **Dyslipidemia** | **5755 (32.6%)** | **4942 (31%)** | **813 (47.3%)** | **<.001** |
| **Malignant cancer** | **1090 (6.2%)** | **991 (6.2%)** | **99 (5.8%)** | **0.48** |
| **Sepsis** | **9543 (54.1%)** | **8288 (52%)** | **1255 (73%)** | **<.001** |
| **Charlson,(median [IQR])** | **4.0 (2.0 to 6.0)** | **4.0 (2.0 to 6.0)** | **5.0 (4.0 to 7.0)** | **<.001** |
| **Laboratory tests** |  |  |  |  |
| **WBC,K/uL,(median [IQR])** | **11.1 (8.2 to 14.8)** | **10.9 (8.1 to 14.7)** | **12.1 (9.0 to 16.1)** | **<.001** |
| **RBC,m/uL,(median [IQR])** | **3.6 (3.1 to 4.2)** | **3.7 (3.2 to 4.2)** | **3.4 (3.0 to 3.9)** | **<.001** |
| **Hemoglobin,g/dL,(median[IQR])** | **11.0 (9.5 to 12.6)** | **11.1 (9.5 to 12.7)** | **10.4 (9.2 to 11.8)** | **<.001** |
| **Initialbloodglucose,mg/dL,(median[IQR])** | **122.3 (105.3 to 143.7)** | **122.0 (105.0 to 143.0)** | **125.7 (108.0 to 147.9)** | **<.001** |
| **Meanbloodglucose,mg/dL,(median[IQR])** | **120.5 (107.2 to 136.0)** | **120.0 (106.4 to 135.7)** | **124.3 (113.2 to 138.3)** | **<.001** |
| **Potassium,mEq/L,(median [IQR])** | **4.1 (3.8 to 4.4)** | **4.1 (3.8 to 4.4)** | **4.3 (4.0 to 4.6)** | **<.001** |
| **Sodium,mEq/L,(median [IQR])** | **138.8 (136.3 to 141.0)** | **138.8 (136.3 to 141.0)** | **139.0 (136.2 to 141.0)** | **0.762** |
| **BUN,mg/dL, (median [IQR])** | **16.5 (11.5 to 25.0)** | **16.0 (11.0 to 24.7)** | **20.0 (14.7 to 30.0)** | **<.001** |
| **SCR,mg/dL, (median [IQR])** | **0.9 (0.7 to 1.2)** | **0.9 (0.7 to 1.2)** | **1.0 (0.8 to 1.4)** | **<.001** |
| **Glucose variability,%,(median[IQR])** | **17.1 (11.5 to 24.4)** | **16.9 (11.3 to 24.2)** | **18.7 (13.4 to 26.2)** | **<.001** |
| **Medications,n(%)** |  |  |  |  |
| **ACEI** | **2617 (14.8%)** | **2325 (14.6%)** | **292 (17%)** | **0.009** |
| **ARB** | **763 (4.3%)** | **672 (4.2%)** | **91 (5.3%)** | **0.044** |
| **Betablocker** | **9139 (51.8%)** | **7663 (48.1%)** | **1476 (85.9%)** | **<.001** |
| **CCB** | **2268 (12.9%)** | **2032 (12.8%)** | **236 (13.7%)** | **0.271** |
| **Digoxin** | **204 (1.2%)** | **77 (0.5%)** | **127 (7.4%)** | **<.001** |
| **Loopdiuretics** | **8009 (45.4%)** | **6651 (41.8%)** | **1358 (79%)** | **<.001** |
| **OAC** | **2460 (13.9%)** | **1740 (10.9%)** | **720 (41.9%)** | **<.001** |
| **Statin** | **1590 (9%)** | **1332 (8.4%)** | **258 (15%)** | **<.001** |
| **Antiplatelet** | **5823 (33%)** | **4793 (30.1%)** | **1030 (59.9%)** | **<.001** |
| **APSIII,(median [IQR])** | **38.0 (29.0 to 52.0)** | **38.0 (28.0 to 51.0)** | **45.0 (34.0 to 61.0)** | **<.001** |
| **OASIS,(median [IQR])** | **31.0 (25.0 to 37.0)** | **30.0 (25.0 to 36.0)** | **35.0 (30.0 to 40.5)** | **<.001** |
| **Length of stay(LOS)** |  |  |  |  |
| **LOS in hospital,day,(median [IQR])** | **8.0 (5.0 to 13.8)** | **7.8 (4.8 to 13.4)** | **10.9 (7.1 to 16.9)** | **<.001** |
| **LOS in ICU,day,(median [IQR])** | **3.0 (2.0 to 5.2)** | **2.9 (1.9 to 4.9)** | **5.2 (3.3 to 9.1)** | **<.001** |

Categorical variables are presented as n (%) and continuous variables are presented as mean (standard deviation). p < 0.05 is taken as statistical significance. Abbreviations: AF, atrial fibrillation; ICU, intensive care unit; CCU, coronary care unit; BMI, body mass index; HR, heart rate; RR, respiratory rate; BP, blood pressure; SBP, systolic blood pressure; DBP, diastolic blood pressure; WBC, white blood cell; RBC, red blood cell; BUN, blood urea nitrogen; SCR, serum creatinine; ACEI, angiotensin converting enzyme inhibitor; ARB, angiotensin receptor blockers; CCB, calcium channel blocker; OAC, oral anticoagulant; APSIII, Acute Physiology Score Ⅲ; OASIS, oxford acute severity of illness score; LOS, length of stay.

**Supporting file, Table S3 Association Between Glucose Variability and New-Onset AF in the four groups according to CV quartiles**

| Groups | Crude OR | Crude  p value | Adjusted OR | Adjusted  p value | Adjusted OR | Adjusted  p value | Adjusted OR | Adjusted  p value | Adjusted OR | p value |
| --- | --- | --- | --- | --- | --- | --- | --- | --- | --- | --- |
|  | Model1 | Model1 | Model2 | Model2 | Model3 | Model3 | Model4 | Model4 | Model5 | Model5 |
| Q1 | ref | - | ref | - | ref | - | ref | - | ref | - |
| Q2 | 1.62 (1.39-1.89) | <0.001 | 1.60 (1.37-1.88) | <0.001 | 1.44 (1.23-1.70) | <0.001 | 1.44 (1.22-1.69) | <0.001 | 1.30 (1.09-1.55) | 0.003 |
| Q3 | 1.92 (1.65-2.24) | <0.001 | 1.90 (1.63-2.22) | <0.001 | 1.59 (1.35-1.86) | <0.001 | 1.57 (1.34-1.85) | <0.001 | 1.43 (1.20-1.70) | <0.001 |
| Q4 | 1.87 (1.60-2.18) | <0.001 | 1.88 (1.61-2.20) | <0.001 | 1.39 (1.18-1.65) | <0.001 | 1.38 (1.16-1.63) | <0.001 | 1.36 (1.13-1.63) | 0.001 |
| P for trend | - | <0.001 | - | <0.001 | - | <0.001 | - | <0.001 | - | 0.001 |

Model1:unadjusted;

Model2: adjusted for Age, Gender, BMI;

Model3: adjusted for Age, Gender, BMI, HR, SBP, DBP, APSⅢ, OASIS, WBC, Hemoglobin, Scr, BUN, Potassium, Mean.blood.glucose;

Model4: adjusted for Age, Gender, BMI, HR, SBP, DBP, APSⅢ, OASIS, WBC, Hemoglobin, Scr, BUN, Potassium, Mean.blood.glucose, Charlson, Hypertension, Dyslipidemia, AMI, Heartfailure, CKD, Chronic pulmonary disease;

Model5: adjusted for Age, Gender, BMI, HR, SBP, DBP, APSⅢ, OASIS, WBC, Hemoglobin, Scr, BUN, Potassium, Mean.blood.glucose, Charlson, Hypertension, Dyslipidemia, AMI, Heartfailure, CKD, Chronic pulmonary disease, ARB, ACEI, betablocker, Digoxin, LoopDiuretics, OAC, Antiplatelet, Statin.

Abbreviations: BMI, Body mass index; HR, Heart rate; SBP, Systolic blood pressure; DBP, Diastolic blood pressure; APSⅢ, Acute Physiology Score III; OASIS, Oxford Acute Severity of Illness Score; WBC, White blood cell; Scr, Serum creatinine; BUN, Blood urea nitrogen; AMI, acute myocardial infarction; CKD, chronic kidney disease; ARB, Angiotensin receptor blockers; ACEI, Angiotensin converting enzyme inhibitors; OAC, Oral anticoagulants.

**Supporting file, Table S4 Association Between Glucose Variability andIn-Hospital Mortality in the four groups according to CV quartiles**

| Groups | Crude OR | Crude pvalue | Adjusted OR | Adjusted  p value | Adjusted OR | Adjusted  p value | Adjusted OR | Adjusted  p value | Adjusted OR | p value |
| --- | --- | --- | --- | --- | --- | --- | --- | --- | --- | --- |
|  | Model1 | Model1 | Model2 | Model2 | Model3 | Model3 | Model4 | Model4 | Model5 | Model5 |
| Q1 | ref | - | ref | - | ref | - | ref | - | ref | - |
| Q2 | 1.44 (1.25-1.66) | <0.001 | 1.42 (1.23-1.64) | <0.001 | 1.08 (0.93-1.26) | 0.335 | 1.09 (0.93-1.28) | 0.2745 | 1.10 (0.94-1.29) | 0.244 |
| Q3 | 1.89 (1.65-2.17) | <0.001 | 1.85 (1.61-2.12) | <0.001 | 1.14 (0.98-1.32) | 0.092 | 1.14 (0.98-1.33) | 0.090 | 1.16 (0.99-1.36) | 0.069 |
| Q4 | 3.33 (2.93-3.80) | <0.001 | 3.26 (2.86-3.71) | <0.001 | 1.33 (1.14-1.54) | <0.001 | 1.38 (1.18-1.61) | <0.001 | 1.37 (1.17-1.61) | <0.001 |
| Groups | - | <0.001 | - | <0.001 | - | <0.001 | - | <0.001 | - | <0.001 |

Model1:unadjusted;

Model2: adjusted for Age, Gender, BMI;

Model3: adjusted for Age, Gender, BMI, HR, SBP, DBP, APSⅢ, OASIS, WBC, Hemoglobin, Scr, BUN, Potassium, Mean.blood.glucose;

Model4: adjusted for Age, Gender, BMI, HR, SBP, DBP, APSⅢ, OASIS, WBC, Hemoglobin, Scr, BUN, Potassium, Mean.blood.glucose, Charlson, Hypertension, Dyslipidemia, AMI, Heartfailure, CKD, Chronic pulmonary disease;

Model5: adjusted for Age, Gender, BMI, HR, SBP, DBP, APSⅢ, OASIS, WBC, Hemoglobin, Scr, BUN, Potassium, Mean.blood.glucose, Charlson, Hypertension, Dyslipidemia, AMI, Heartfailure, CKD, Chronic pulmonary disease, ARB, ACEI, betablocker, Digoxin, LoopDiuretics, OAC, Antiplatelet, Statin.

Abbreviations: BMI, Body mass index; HR, Heart rate; SBP, Systolic blood pressure; DBP, Diastolic blood pressure; APSⅢ, Acute Physiology Score III; OASIS, Oxford Acute Severity of Illness Score; WBC, White blood cell; Scr, Serum creatinine; BUN, Blood urea nitrogen; AMI, acute myocardial infarction; CKD, chronic kidney disease; ARB, Angiotensin receptor blockers; ACEI, Angiotensin converting enzyme inhibitors; OAC, Oral anticoagulants.

**Supporting file, Table S5 Direct and indirect effects of glycemic** variability on in-hospital death

|  | Full association, OR(95%CI) | Direct association, OR(95%CI) | Mediated by AF, OR(95%CI) | Percent mediation, % | P value for the indirect effect |
| --- | --- | --- | --- | --- | --- |
| Crude | 1.47(1.42–1.52) | 1.42(1.37–1.47) | 1.04(1.03–1.05) | 9.35 | <0.001 |
| Adjusted | 1.07(1.02–1.13) | 1.05(0.99–1.1) | 1.03(1.01–1.04) | 36.26 | <0.001 |

Adjusted for gender, age, race, BMI, ICU category, heart rate, respiratory rate, WBC, hemoglobin, Scr, potassium, sodium, chronic pulmonary disease, cerebrovascular disease, hypertension, heart failure, chronic kidney disease, dyslipidemia, malignant cancer, sepsis, medications and disease severity scores( Charlson, APSⅢ, and OASIS).

Abbreviations: AF, atrial fibrillation; ICU, intensive care unit; CCU, coronary care unit; BMI, body mass index; WBC, white blood cell; BUN, blood urea nitrogen; SCR, serum creatinine; APSⅢ, Acute Physiology ScoreⅢ ; OASIS, oxford acute severity of illness score.

**Supporting file, Table S6 Subgroup Mediation Analysis**

| **Variable** | **Subgroup** | **Indirect Effect OR**  **(95% CI)** | **P value** | **P for interaction** |
| --- | --- | --- | --- | --- |
| Age | Age < 65 | 1.01 (1.00, 1.01) | 0.119 |  |
| Age | Age ≥ 65 | 1.01 (1.01, 1.02) | <0.001 | 0.194 |
| BMI | BMI < 30 | 1.01 (1.00, 1.02) | 0.004 |  |
| BMI | BMI ≥ 30 | 1.01 (1.00, 1.02) | 0.125 | 0.781 |
| Gender | Gender (Female) | 1.01 (1.00, 1.02) | 0.003 |  |
| Gender | Gender (Male) | 1.01 (1.00, 1.01) | 0.031 | 0.167 |
| Hypertension | Hypertension (No) | 1.01 (1.00, 1.02) | 0.002 |  |
| Hypertension | Hypertension (Yes) | 1.01 (1.00, 1.01) | 0.065 | 0.134 |
| AMI | AMI (No) | 1.01 (1.00, 1.02) | 0.001 |  |
| AMI | AMI (Yes) | 1.01 (1.00, 1.02) | 0.122 | 0.966 |
| Heartfailure | Heartfailure (No) | 1.01 (1.00, 1.02) | 0.003 |  |
| Heartfailure | Heartfailure (Yes) | 1.01 (1.00, 1.02) | 0.046 | 0.734 |
| CKD | CKD (No) | 1.01 (1.00, 1.01) | 0.006 |  |
| CKD | CKD (Yes) | 1.03 (1.01, 1.05) | 0.010 | 0.064 |
| Dyslipidemia | Dyslipidemia (No) | 1.01 (1.00, 1.02) | 0.003 |  |
| Dyslipidemia | Dyslipidemia (Yes) | 1.01 (1.00, 1.02) | 0.064 | 0.418 |
| Sepsis | Sepsis (No) | 1.00 (1.00, 1.01) | 0.520 |  |
| Sepsis | Sepsis (Yes) | 1.01 (1.01, 1.02) | <0.001 | 0.028 |
| Race | Race (ASIAN) | 1.00 (0.99, 1.01) | 0.996 |  |
| Race | Race (BLACK) | 1.08 (1.00, 1.16) | 0.048 |  |
| Race | Race (OTHER) | 1.01 (0.98, 1.04) | 0.564 |  |
| Race | Race (UNKNOWN) | 1.01 (0.99, 1.03) | 0.328 |  |
| Race | Race (WHITE) | 1.01 (1.00, 1.01) | 0.010 |  |
| Chronic pulmonary disease | Chronic pulmonary disease (No) | 1.01 (1.00, 1.01) | 0.001 |  |
| Chronic pulmonary disease | Chronic pulmonary disease (Yes) | 1.03 (0.98, 1.08) | 0.215 | 0.380 |
| Cerebrovascular disease | Cerebrovascular disease(No) | 1.01 (1.00, 1.01) | 0.002 |  |
| Cerebrovascular disease | Cerebrovascular disease(Yes) | 1.01 (0.99, 1.03) | 0.233 | 0.832 |
| Malignantcancer | Malignantcancer (No) | 1.01 (1.00, 1.01) | 0.002 |  |
| Malignantcancer | Malignantcancer (Yes) | 1.06 (1.01, 1.11) | 0.023 | 0.052 |

All models were adjusted for covariates such as demographic characteristics, vital signs, laboratory indicators and complications (excluding stratified variables themselves). P for interaction was only calculated for the binary variables which can obtain the estimations of two subgroups of effective models at the same time.

Path a: the influence the LogCV on the risk of AF;

Path b: the influence of AF on the risk of hospital death;

Indirect Effect: defined as the product of path coefficients (a×b), the result is converted into odds ratio (OR) and its 95% confidence interval for easy explanation.

Abbreviations: BMI, body mass index; AMI, acute myocardial infarction; CKD, chronic kidney disease; AF, atrial fibrillation.
